# Culture filtrate supplementation can be used to improve *Mycobacterium tuberculosis* culture positivity for spinal tuberculosis diagnosis

**DOI:** 10.3389/fcimb.2022.1065893

**Published:** 2022-11-25

**Authors:** Caroline G. G. Beltran, Rouxjeane Venter, Theresa N. Mann, Johan H. Davis, Bavesh D. Kana, Gerhard Walzl

**Affiliations:** ^1^ Department of Science and Technology-National Research Foundation (DST-NRF) Centre of Excellence for Biomedical Tuberculosis Research, South African Medical Research Council Centre for Tuberculosis Research, Division of Molecular Biology and Human Genetics, Faculty of Medicine and Health Sciences, Stellenbosch University, Cape Town, South Africa; ^2^ Division of Orthopaedic Surgery, Department of Surgical Sciences, Faculty of Medicine and Health Sciences, Stellenbosch University, Cape Town, South Africa; ^3^ Department of Science and Technology-National Research Foundation (DST-NRF) Centre of Excellence for Biomedical TB Research, School of Pathology, Faculty of Health Sciences, University of the Witwatersrand and the National Health Laboratory Service, Johannesburg, South Africa; ^4^ Medical Research Council Centre for the Aids Programme of Research in South Africa (MRC-CAPRISA) HIV-TB Pathogenesis and Treatment Research Unit, Centre for the AIDS Programme of Research in South Africa, CAPRISA, Durban, South Africa

**Keywords:** spinal TB, extrapulmonary TB, culture, dormancy, resuscitation - methods, diagnostic

## Abstract

Culture remains the gold standard to diagnose spinal tuberculosis (STB) despite the paucibacillary nature of the disease. Current methods can take up to 42 days to yield a result, delaying the ability to rapidly detect drug resistance. Studies have demonstrated the use of supplementation with culture filtrate (CF) from an axenic culture of *Mycobacterium tuberculosis* (*Mtb*) as a source of growth factors to improve culture rates. Our objective was to test a modified culture assay, utilizing CF supplemented media (CFSM), to improve culture positivity rates for suspected STB. Twelve patients with suspected STB were assessed by conventional culture (BACTEC™ MGIT 960), GeneXpert™ and standard histopathological examination. Spinal biopsies were taken from areas of diseased vertebral tissue or abscess, predetermined from MRI. Additional biopsies were obtained to assess CFSM for improved detection and faster culture of Mtb. All cases were diagnosed as STB and treated empirically for tuberculosis based on either bacteriological evidence (GeneXpert™, MGIT and/or CFSM positive), or based on clinical presentation. 5 specimens (45.45%) were positive for *Mtb* DNA as detected by GeneXpert™ and 1 specimen (8.33%) was cultured using MGIT (time to detection; 18 days). CFSM was able to culture 7 specimens (58.3%), with all CFSM positive specimens yielding a culture within 14 days. Two samples were positive only using the CFSM assay pointing to additional yield for diagnostic workup. Modification of standard culture can improve detection of *Mtb* and reduce time to positivity in individuals with STB where culture material is a requirement.

## Introduction

Diagnosis of spinal tuberculosis (STB), an extra pulmonary tuberculosis (EPTB) disease, remains difficult due to the slow, insidious onset of symptoms, variation in clinical presentation and health system delays (Chen et al., 2016). STB accounts for ~50% of musculoskeletal-TB and involves the progressive destruction of spinal vertebrae (Garg and Somvanshi, 2011). While a combination of radiological and clinical findings are used to establish diagnosis, culturing of the etiological agent remains the gold standard (Agrawal et al., 2010). However, culture positivity rates for STB can vary and may take as long as 42 days (Agrawal et al., 2010; Colmenero et al., 2013; Watt and Davis, 2014; Mann et al., 2018), potentially contributing to treatment delays and poorer outcomes.

Given the concerns associated with standard culture, molecular diagnostics such as the GeneXpert™

MTB/RIF (GeneXpert™), are becoming routinely used for diagnosis of EPTB. However,

GeneXpert™ also has limitations. Reduced sensitivity for paucibacillary samples has been reported and test results may be confounded by DNA from dead bacilli, an important consideration for the assessment of treatment response (Kohli et al., 2018). Furthermore, at least 5% of STB cases in our setting have multi- and extensively-drug resistant STB, requiring further methods for drug susceptibility testing (DST). Line probe assays including the Genotype MTBDR*plus* and MTBDR*sl* (HAIN Lifesciences, Germany) can detect resistance to first– and second-line drugs, yet suffer from poor sensitivity in paucibacillary samples. Thus, it is recommended that these assays are done on cultured isolates for definitive DST (Kumari et al., 2016).

While the paucibacillary nature of the sample may contribute to poor culture rates, altered physiological growth states of *Mtb*, such as a dormancy related states, add another layer of complexity. The ability for *Mtb* to switch to a non-replicating state during periods of stress is well characterized and indicates a hidden population which are differentially culturable (DC), and thus undetectable, on routine culture mediums (Mukamolova et al., 2003). Recent studies have demonstrated the presence of these DC cells in sputa of TB patients (Mukamolova et al., 2010; Chengalroyen et al., 2016; Rosser et al., 2018; Beltran et al., 2020). These cells appear to be dependent on supplementation by culture filtrate (CF), sourced from actively growing *Mtb* cultures, to resume their growth. CF supplementation has been shown to reduce lag time of liquid culture and resuscitate populations of dormant *Mtb* which were otherwise undetectable by standard culture (Chengalroyen et al., 2016; Beltran et al., 2020).

The growth stimulatory effect of CF is primarily attributed to a family of lytic transglycosylase-like proteins, known as resuscitation promoting factors (RPFs), thought to cleave the cell wall of dormant cells and allowing division to resume (Kana and Mizrahi, 2010). Although RPFs have been demonstrated to play a key role in uncovering dormant cells, *Mtb* CF has been shown to have superior growth-stimulatory activity compared to recombinant RPF alone, most likely due to the biological stability of RPFs in CF (Chengalroyen et al., 2016).

The use of CF to culture EPTB samples is limited and has only been conducted in two studies (O’Connor et al., 2015; Gleeson et al., 2016). Conflicting results could not definitively demonstrate the utility of CF to increase culture detection for EPTB, possibly in part be due to differences in the sample collection (freshly acquired vs frozen). The utility of CF supplementation, and presence of altered *Mtb* cells in various EPTB samples, thus warrants further investigation.

Here, we report the use of a modified-culture assay, using CF supplementation, to investigate the utility of this approach in improving culture positivity rates for STB.

## Materials and methods

### Participant recruitment and sample collection

The study was approved by the Health Research Ethics Committee of Stellenbosch University (N16/02/029) and by Tygerberg Hospital. Treatment-naïve participants, > 18 years of age (8 men and 4 women, age mean ± standard deviation 42 ± 20 years), with clinical and radiological signs suggestive of STB were approached and provided written, informed consent prior to undergoing a diagnostic spinal biopsy.

Depending on the location of the lesion, percutaneous transpedicular core needle biopsies were performed in the lumbar -and thoracolumbar spine, with a small open costotransversectomy approach typically utilized in the thoracic spine, in order to gain access to the paravertebral abscess and diseased tissue. Spinal tissue biopsies were collected from the same diseased area under strict aseptic conditions, and placed into separate containers containing sterile saline for simultaneous processing. As far as possible, equal material was collected and sent for the following: 1) routine diagnostic pathology (haematoxylin and eosin stain), 2) standard liquid culture (BACTEC™ MGIT 960), 3) GeneXpert™ testing and finally, 4) CFSM assay. A trained pathologist experienced in reviewing and diagnosing TB reviewed the histopathological slides. Criteria for TB included caseous necrosis, presence of granulomas/granulomatous regions, epitheloid cells and Langerhans giant cells.The automated liquid BACTEC™ MGIT 960 by BD Diagnostic Systems, Sparks MD (Franklin Lakes, New Jersey) was used for standard culture. Tissue biopsies were processed according to the manufacturer’s instructions. Briefly, tissue biopsies were agitated *via* vortexing and removed from the sterile saline using forceps and placed inside a MGIT tube containing reconstituted antimicrobial mixture PANTA™ (Polymyxin B, Amphotericin B, Nalidixic Acid, Trimethoprim, Azlocillin). Tubes were placed inside a BACTEC™ MGIT 960 by BD Diagnostic System and monitored over a period of 42 days for growth.

GeneXpert™ testing was performed according to the manufacturer’s instructions. Briefly, tissue biopsies were transferred to a 15mL falcon tube and 2mL lysis buffer was added to the sample and vortexed periodically during the 15-minute incubation period at room temperature. Following which, 2mL of the material was transferred into the cartridges and loaded into the machine for automated testing.

### Preparation of culture filtrate supplemented media

A laboratory strain of *Mtb*, H37Rv, was used to produce CF and was prepared as described previously (Shleeva et al., 2002). Fresh 7H9 media was supplemented with CF (1:1) and PANTA antibiotic mixture (BD Biosciences). A 450 µL aliquot of the culture filtrate supplemented media (CFSM) was added to a 48-well multidish cell culture plate (Thermo Scientific Nunc). Neat CFSM aliquots were included as controls.

### Addition of spinal biopsies to modified culture assay

Spinal biopsies were prepared for the CFSM assay within 24 hours of collection. Biopsies were removed aseptically from the collection container and transferred to a well containing 450 µL of CFSM. Culture plates were sealed with micropore-tape and incubated at 37°C for 8 weeks and checked for growth weekly. A positive well was determined visually, through development of turbidity, and presence of *Mtb* was subsequently confirmed [Ziehl-Neelsen staining, single colony isolation on Mycobacteria Selectatab media (Kirchner) and strain typed using spoligotyping (Molhuizen et al., 1998)]. A negative culture well was defined as one in which neither *Mtb* grew, nor contamination was observed.

## Results

### Assessment of modified culture assay for spinal biopsies

In total, 12 patients were assessed for spinal TB diagnosis using standard pathology, liquid culture

(BACTEC™ MGIT 960), GeneXpert™ and the CFSM assay. All cases were diagnosed as STB and treated empirically for tuberculosis based on either bacteriological evidence (GeneXpert™ and/or MGIT and/or CFSM positive) or based on clinical presentation.

Of the 12 patients assessed, 4 (33.3%) had negative bacteriological spine-biopsy findings and were diagnosed based on clinical presentation alone, including spine MRI suggestive of TB (**Table 1**).

**Table 1:** Suspected spinal TB cases (n=12) assessed by GeneXpert™, BACTEC™ MGIT 960, modified culture (CFSM) and/or histology.

PID	GeneXpert	MGIT (TTD)	CFSM assay (TTD)	Strain type	Histology	STB diagnosis
SP003	+	+ (18)	+ (11)	Beijing 1	ND	Bact.
SP002	+	–	+ (3)	156T1	No gran. infl.	Bact.
SP029	ND	–	+ (14)	LAM 33	Inconclusive	Bact.
SP031	–	–	+ (12)	Beijing 1	Chronic infl.	Bact.
SP037	+	–	+ (9)	LAM 33	Diffuse gran. infl.	Bact.
SP038	–	–	+ (11)	130 LAM 3	Necrotic bone, chronic infl.	Bact.
SP041	+	–	+ (12)	Beijing 1	Necrosis and acute infl.	Bact.
SP028	+	–	–	NA	Gran. infl.	Bact.
SP034	–	–	–	NA	Chronic infl.	Clin.
SP036	–	–	–	NA	Hypocellular bone, no gran. infl.	Clin.
SP046	–	–	–	NA	Osteitis, no gran. infl.	Clin.
SP047	–	–	–	NA	Necrotic material, no gran. infl.	Clin.
Percentage detected	45.45%	8.33%	58.3%			

(PID) patient identifier; (+) positive result; (-) negative result; (TTD) time to detection in days; (ND) not done; 257 (STB) Spinal Tuberculosis; (gran.) granulomatous; (infl.) inflammation; (Bact.) bacteriological; (Clin.) clinical; 258 (NA) Not Applicable; TTD (time to detection) shown in days for culture (MGIT 254 and CFSM). Strain type refers to the genotyping of the strains recovered by the modified culture 255 assay.

**Figure 1:**
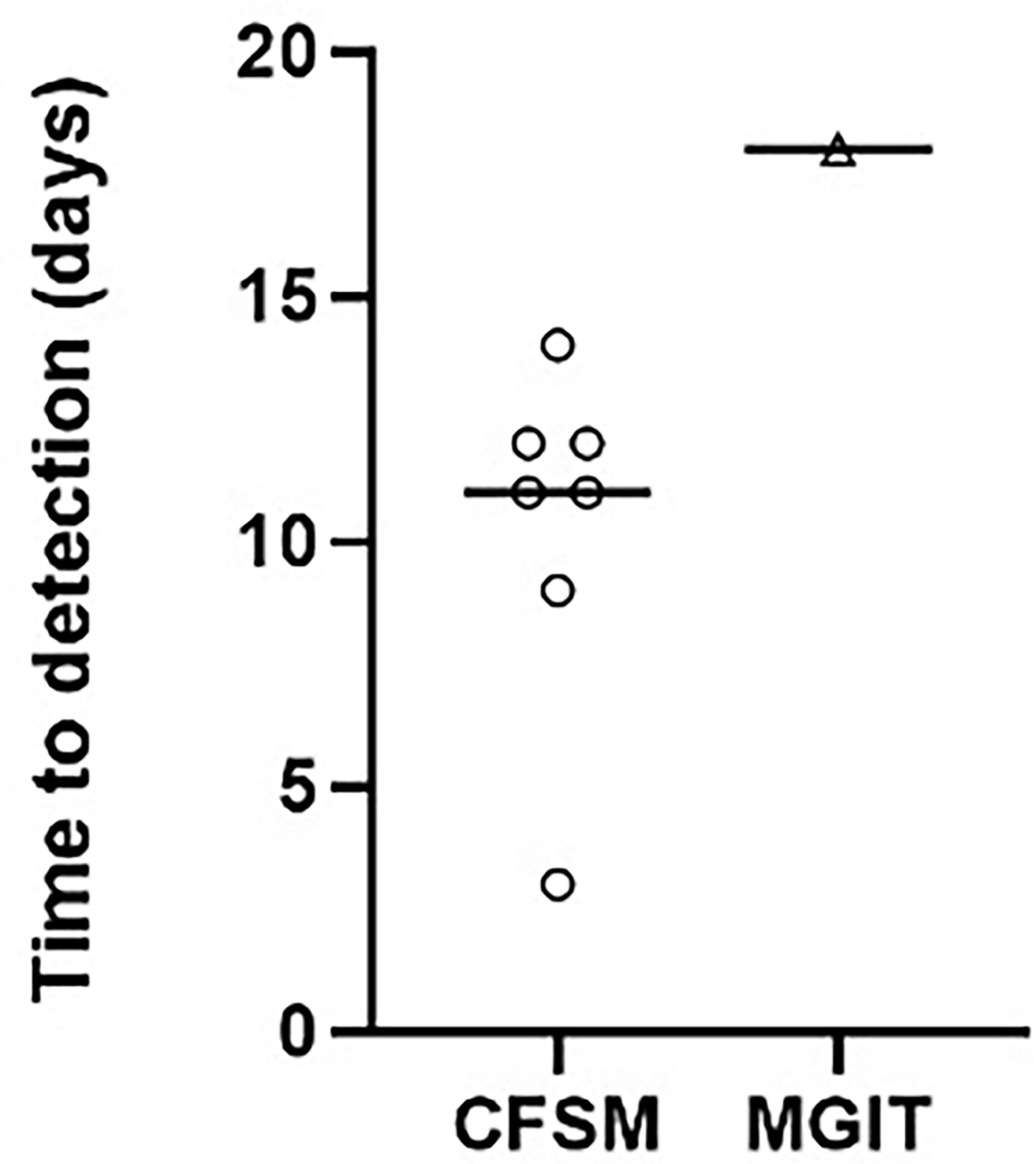
Scatter plot comparing the time to detection in days for positive cultures of *M. tuberculosis* from spinal biopsies using culture filtrate supplemented media vs standard liquid culture (Mycobacteria Indicator Growth Indicator Tube; MGIT). A negative culture result where no growth was detected was assigned as negative and not displayed on the graph. Median time to detection was 11 and 18 days for CFSM and MGIT culture, respectively.

The remaining 8 patients (66.67%) could be diagnosed based on a bacteriological diagnosis (**Table 1**). *Mtb* DNA was detected in 5 samples (45.45%) using GeneXpert™, with one sample not being sent for analysis. Standard culture (BACTEC™ MGIT) only detected 1 sample (8.33%), with a time to positivity of 18 days (**Figure 1**), all other samples remained culture negative. The CFSM assay detected 7 positive samples (58.3%), with a median time to detection of 9 days (**Figure 1**). Compared to GeneXpert™, CFSM was able to detect 2 samples as *Mtb* positive that were negative by GeneXpert™, whilst one sample was positive for GeneXpert only (**Table 1**).

## Discussion

In this study, we have shown that a modified culture assay of spinal biopsies utilizing CFSM can be used to increase the culture yield and time-to-positivity for *Mtb* and could add value to the diagnostic workup of suspected STB, in conjunction with GeneXpert™. The positive STB samples all yielded detectable growth within 2 weeks using the CFSM assay. These results support an earlier study that used CFSM to culture a lymph node biopsy that was otherwise negative for all other diagnostic tests for TB (O’Connor et al., 2015), although this is in contrast to a later study that found that CF did not increase archived (frozen) specimens (Gleeson et al., 2016). This may indicate the requirement that a fresh sample is required for this assay, which may not always be practical in a clinical setting. This assay does have the advantage of detecting bacteria in a dormancy-related state which may be clinically relevant (Chengalroyen et al., 2016; Beltran et al., 2020; Gordhan et al., 2021). One of the main limitations of this assay is the requirement of fresh preparation of CF, which is laborious for a clinical laboratory to implement, though standardization of CF as a supplement to add to current MGIT tubes could be developed. Overall, the CFSM assay correlated well with GeneXpert™ results and performed better than standard MGIT culture. Two samples were positive only for the CFSM assay pointing to additional yield for diagnostic workup since further DST using the MTBDR*plus* and MTBDR*sl* LPA’s can be done on the cultured isolate (Kumari et al., 2016).

These conclusions are limited by the assessment of the CFSM assay in a small cohort of participants however, whilst further investigation is needed, these results provide strong support that this assay could be applied to paucibacillary sample types where culture has proven difficult and can lead to earlier detection of *Mtb*.

## Data availability statement

The original contributions presented in the study are included in the article/supplementary material. Further inquiries can be directed to the corresponding author.

## Ethics statement

The studies involving human participants were reviewed and approved by Health Research Ethics Committee of Stellenbosch University. The patients/participants provided their written informed consent to participate in this study.

## Author contributions

All authors listed, have made substantial, direct and intellectual contribution to the work, and approved it for publication. CB, TM, JD and GW contributed to the conception and design of the study. CB, RV and TM collected data, analyzed the results and wrote the manuscript. All authors contributed to the article and approved the submitted version.

## Funding

CB was supported by the National Research Foundation South African Research Chair Initiative (SARChI) in TB Biomarkers (#86535) led by GW. GW was supported by the South African Medical Research Council (Strategic Health Innovation Program), the South African National Research Foundation (SARChi, grant 86535) and grant 1U01AI115619 – 01 from the NIH. BK was supported by funding from an International Early Career Scientist Award from the Howard Hughes Medical Institute, the South African National Research Foundation, the South African Medical Research Council and the Bill and Melinda Gates Foundation. TM was supported by postdoctoral fellowships from the National Research Foundation and from the Vice Dean of Research in the Faculty of Medicine and Health Sciences, Stellenbosch University. RV was supported by the National Research Foundation and the Faculty of Medicine and Health Sciences.

## Acknowledgments

We would like to thank all participants who participated in this study.

## Conflict of interest

The authors declare that the research was conducted in the absence of any commercial or financial relationships that could be construed as a potential conflict of interest.

## Publisher’s note

All claims expressed in this article are solely those of the authors and do not necessarily represent those of their affiliated organizations, or those of the publisher, the editors and the reviewers. Any product that may be evaluated in this article, or claim that may be made by its manufacturer, is not guaranteed or endorsed by the publisher.
